# Effects of Noisy Galvanic Vestibular Stimulation on Blood Pressure and Postural Sway in Healthy Adults: A Pilot Study

**DOI:** 10.7759/cureus.94450

**Published:** 2025-10-13

**Authors:** Akiyoshi Matsugi

**Affiliations:** 1 Physical Medicine and Rehabilitation, Shijonawate Gakuen University, Daitō, JPN

**Keywords:** balance control, blood pressure, cardiovascular safety, healthy adults, noisy galvanic vestibular stimulation, postural sway

## Abstract

Background: Noisy galvanic vestibular stimulation (nGVS) has been shown to influence postural control, but its cardiovascular safety remains uncertain. Establishing tolerability and hemodynamic neutrality is essential for potential clinical use.

Objective: To examine the effects of nGVS on mean blood pressure (MBP) and postural sway in healthy young adults during upright standing.

Methods: Twenty-one healthy volunteers stood with eyes closed on a rubber mat for one minute while receiving sham or real nGVS (1 mA, 40 seconds). Postural sway was recorded with a force plate to obtain total path length (CoP-L), anteroposterior velocity (Vel-AP), and mediolateral velocity (Vel-ML). MBP was measured immediately after each trial with an automated sphygmomanometer. Outcomes were normalized to baseline, averaged within participants, and compared between conditions using paired analyses. Mixed-effects models explored associations between MBP and sway velocity.

Results: All participants reported no pain or dizziness (numeric rating scale, or NRS = 0), confirming tolerability. No significant differences were observed between sham and real nGVS for MBP or sway parameters (all p > 0.05, Cohen’s dz < 0.4). Exploratory analyses suggested a weak correlation between MBP and Vel-ML under real nGVS, but this association was not significant when repeated measures were modeled.

Conclusions: Brief 1 mA nGVS was imperceptible, safe, and produced no detectable changes in blood pressure or sway in healthy young adults. These findings provide preliminary evidence of cardiovascular safety and support further studies in clinical populations at risk of autonomic dysfunction or balance impairment.

## Introduction

Galvanic vestibular stimulation (GVS) is a non-invasive neuromodulation technique that delivers weak electrical currents through electrodes placed over the mastoid processes to alter vestibular input [1]. In recent years, noisy galvanic vestibular stimulation (nGVS), which applies Gaussian white noise, has gained attention for its ability to enhance vestibular function through stochastic resonance mechanisms [2]. Several studies have demonstrated that nGVS can reduce postural sway and improve balance in elderly individuals and in patients with vestibular disorders [3-7], suggesting its potential as a safe and effective intervention in clinical rehabilitation.

Beyond its role in maintaining postural stability, the vestibular system contributes to autonomic regulation via projections to brainstem centers that influence cardiovascular activity. Experimental studies using sinusoidal or pulse-type GVS have shown that vestibular stimulation can induce changes in arterial pressure and heart rate variability, highlighting a vestibulo-autonomic connection [8]. However, the specific effects of nGVS on cardiovascular responses, such as blood pressure, remain largely unclear. Establishing the hemodynamic safety profile of nGVS is crucial if it is to be applied more widely in neurorehabilitation practice.

The stimulation delivers imperceptible stochastic electrical currents to vestibular afferents, enhancing neural signal detection through stochastic resonance. By modulating vestibulospinal and vestibulo-autonomic pathways, nGVS may influence postural control and cardiovascular regulation, which could underlie its reported effects on balance and hemodynamic function.

Recent studies have begun to elucidate the broader physiological effects of nGVS. We previously reported that nGVS modulates body sway and lower-limb muscle activity in healthy young adults [9], does not affect arterial pressure or heart rate variability in elderly participants [10], and alters vestibulo-ocular reflex gain in young adults [11]. Other groups have also expanded the evidence base: Fujimoto and colleagues demonstrated that nGVS can improve balance in both older adults and patients with vestibular disorders [12]; Inukai et al. showed that the effectiveness of nGVS may depend on baseline postural stability [7]; and Mitsutake et al. investigated its application in rehabilitation contexts [13]. Together, these studies support the potential benefits of nGVS while underscoring the need for further safety data.

Therefore, the present pilot study aimed to investigate the effects of nGVS on mean blood pressure (MBP) and postural sway in healthy young adults during quiet upright standing. Additionally, subjective symptoms such as pain and dizziness were monitored to assess tolerability. By clarifying the hemodynamic and postural responses to nGVS in this population, we sought to provide foundational evidence for its safety and to inform future applications in clinical neurorehabilitation.

This study was presented in part at the Annual Meeting of the Japanese Society of Clinical Neurophysiology, 2020 (poster presentation).

## Materials and methods

Participants

Twenty-one healthy young adults (14 men, 7 women; mean age 20.1 ± 0.6 years) without any history of neurological, cardiovascular, or vestibular disorders participated. All participants provided written informed consent prior to participation. The study protocol was approved by the Research Ethics Committee of Shijonawate Gakuen University (approval code: 19-5) and was conducted in accordance with the Declaration of Helsinki.

Experimental procedure

**Figure 1:**
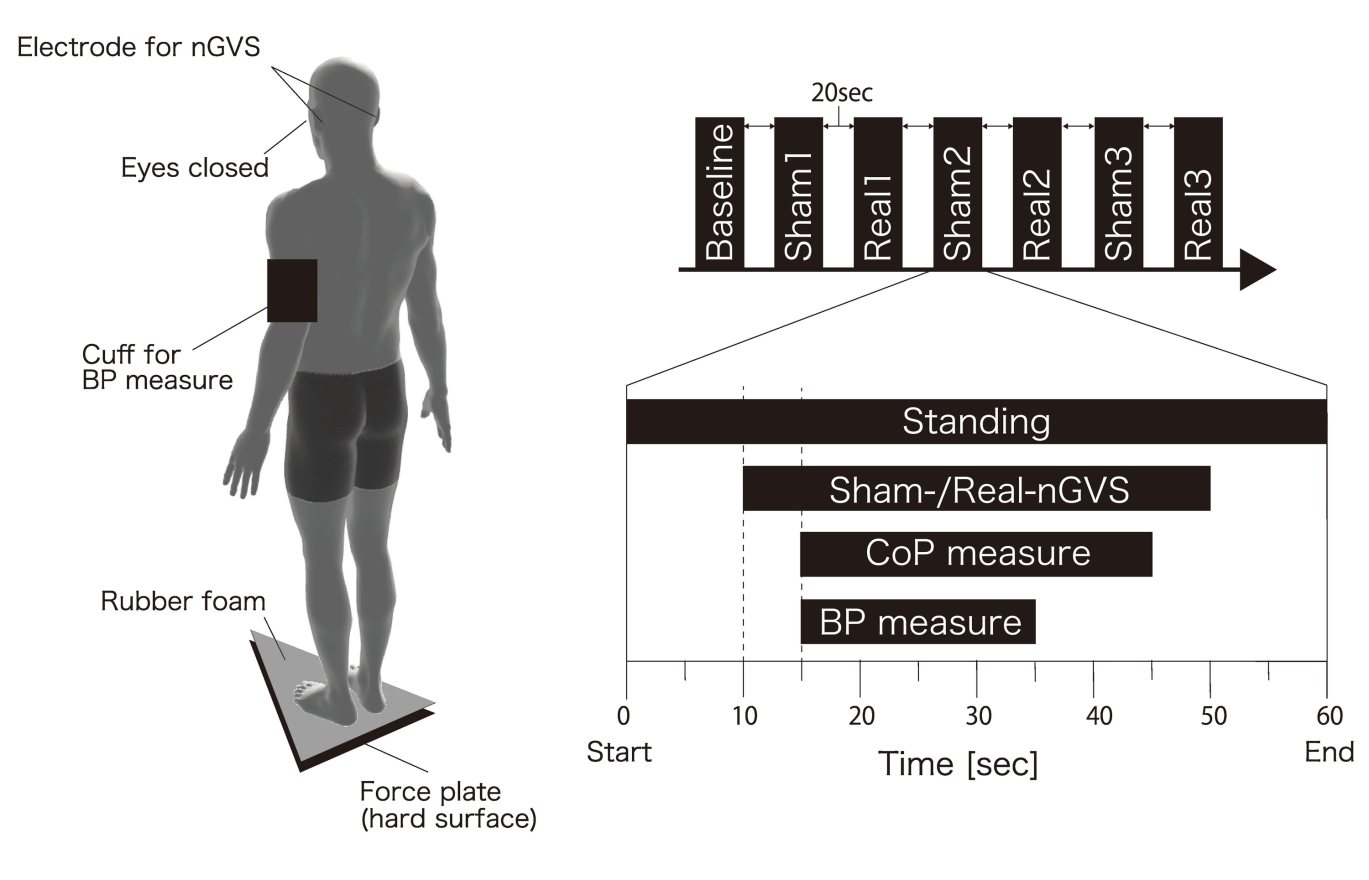
Schematic representation of the experimental procedure Each standing trial lasted 60 seconds, including 40 seconds of sham (0 mA) or real (1 mA) nGVS. Blood pressure was measured immediately after each trial using an automated sphygmomanometer, and postural sway was continuously recorded with a force plate. A 20-second interval was provided between trials while participants remained standing. nGVS: noisy galvanic vestibular stimulation; CoP: center of pressure; BP: blood pressure Image credit: The authors.

Participants stood barefoot with eyes closed on a rubber mat (thickness 3.5 cm; Anima Co., Tokyo, Japan) to reduce somatosensory reliability (Figure 1). They were instructed to maintain an upright posture with feet together and arms at the sides for one minute per trial. The sequence consisted of one baseline trial without stimulation, followed by three sham-nGVS and three real-nGVS trials. Each trial lasted 60 seconds, during which sham or real nGVS (40 seconds) was applied. Between trials, a 20-second inter-trial interval was provided while participants remained standing in the same posture. After each trial, participants rated pain and dizziness/vertigo using a numeric rating scale (NRS, 0-10).

Noisy galvanic vestibular stimulation (nGVS)

nGVS was delivered using a DC-STIMULATOR PLUS (NeuroConn GmbH, Ilmenau, Germany) via 25 × 35 mm Ag/AgCl surface electrodes (Blue Sensor; Ambu, Denmark) placed bilaterally over the mastoid processes. The electrodes were fixed with adhesive tape and moistened sponges after cleaning the skin with alcohol, and skin impedance was kept below 5 kΩ. The real-nGVS condition consisted of Gaussian white noise (0-640 Hz) at 1.0 mA for 40 seconds. In the sham condition, the device was connected, but the current was set to 0 mA. Participants were blinded to the stimulation type; none reported perceiving the stimulation. Data analysts were not informed of condition assignments during statistical processing.

Measurements

Postural sway was recorded using a force plate (Gravicorder G5500; Anima Co.) at 20 Hz. The total trajectory length of the center of pressure (CoP-L), mean velocity in the anteroposterior direction (Vel-AP), and mean velocity in the mediolateral direction (Vel-ML) were calculated. Velocity was derived from point-to-point CoP displacement divided by sampling interval, expressed in cm/s. MBP was measured immediately after each trial using an automated upper-arm sphygmomanometer (HEM-7120; Omron Healthcare, Japan).

Data processing

For each participant, outcomes were normalized to baseline by dividing sham and real values by the individual baseline mean. Values from repeated trials under the same condition were averaged within each participant to yield one sham and one real score per outcome. These subject-level averages were used for the main analyses.

Statistical analysis

Primary comparisons between sham and real conditions were performed using paired t-tests; Wilcoxon signed-rank tests were additionally calculated for robustness. Effect sizes were expressed as Cohen’s dz, and 95% confidence intervals (CIs) for mean differences were reported. Associations between MBP and sway parameters were explored using linear mixed-effects models (MBP as dependent variable, Vel-ML as predictor, random intercept for subject). No corrections for multiple testing were applied, and these analyses were considered exploratory. Statistical significance was set at α = 0.05. All analyses were conducted using JASP (version 0.17.1; JASP Team, Amsterdam, The Netherlands) and Python (statsmodels version 0.14; Python Software Foundation, Wilmington, DE, USA).

## Results

All participants tolerated the procedure without pain, vertigo, or dizziness, and NRS scores remained at 0 throughout. After normalization to baseline, MBP and CoP parameters did not differ significantly between real- and sham-nGVS conditions (Table 1). Consistently, effect sizes were small across all comparisons (Cohen’s dz < 0.4). Exploratory Pearson correlation analyses suggested a weak positive association between MBP and mediolateral sway velocity (Vel-ML), both expressed as baseline-normalized values, under the real-nGVS condition (r = 0.25, p = 0.047). However, this association did not remain significant when repeated measures were modeled using mixed-effects analysis and should therefore be interpreted with caution. No significant associations were observed under sham stimulation or for other sway parameters (Table 2).

**Table 1:** Paired comparisons (real-sham) Outcome: analyzed parameter (normalized if baseline-corrected); n: number of subjects included; MeanDiff(Real-Sham): mean difference between conditions; 95%CI_low/high: confidence interval of mean difference; Paired_t and p_t: paired-samples t-test statistic and p-value; Cohen_dz: standardized effect size for paired samples; Wilcoxon_W and p_w: Wilcoxon signed-rank test statistic and p-value MBP: mean blood pressure; Vel_MP: mediolateral velocity; Vel_AP: anteroposterior velocity

Outcome	n	MeanDiff(Real-Sham)	95%CI_low	95%CI_high	Paired_t	p_t	Cohen_dz	Wilcoxon_W	p_w
MBP_norm	21	-0.017	-0.037	0.003	-1.767	0.092	-0.386	60	0.093
Vel_ML_norm	21	-0.028	-0.086	0.03	-0.993	0.332	-0.217	73	0.147
Vel_AP_norm	21	0.014	-0.043	0.071	0.506	0.618	0.11	111	0.892

**Table 2:** Mixed-effects model (MBP ~ Vel-ML) Condition: stimulation condition (Real or Sham); n_subjects: number of participants; n_points: total data points; Coef_VelML_z: regression coefficient for z-scored Vel-ML predicting MBP; 95%CI_low/high: Wald confidence interval; p_value: significance level for coefficient; Outcome_Y: dependent variable; Predictor_X: independent variable; MBP_norm and Vel_ML_norm indicate values normalized to baseline. MBP: mean blood pressure; Vel_MP: mediolateral velocity; Vel_AP: anteroposterior velocity

Condition	n_subjects	n_points	Coef_VelML_z	95%CI_low	95%CI_high	p_value	Outcome_Y	Predictor_X
Real	21	21	0.022	-0.006	0.05	0.124	MBP_norm	Vel_ML_norm
Sham	21	21	0.007	-0.024	0.039	0.651	MBP_norm	Vel_ML_norm

## Discussion

This pilot study investigated the effects of nGVS on blood pressure and postural sway in healthy young adults during upright standing. The main findings were that (1) nGVS was imperceptible and induced no adverse sensations, confirming its tolerability; and (2) after normalization to baseline and averaging within subjects, no significant differences were detected in MBP or CoP parameters between sham and real stimulation.

Our results are consistent with previous studies demonstrating the safety of nGVS. Fujimoto and colleagues reported that nGVS reduces postural sway in elderly individuals and in patients with vestibular disorders, while Inukai et al. showed that its effects depend on baseline instability. In line with our findings, we previously found that nGVS does not alter arterial pressure or heart rate variability in elderly participants [10]. Collectively, these studies indicate that nGVS is unlikely to induce adverse cardiovascular effects across populations.

Exploratory Pearson correlation initially suggested a weak positive association between MBP and mediolateral sway velocity (r = 0.25, p = 0.047) under the real-nGVS condition. However, this effect did not remain significant when repeated measures were properly modeled using mixed-effects analysis. Therefore, the evidence for a vestibulo-autonomic interaction is currently inconclusive. Larger studies with continuous beat-to-beat blood pressure monitoring will be needed to clarify whether subtle relationships exist between cardiovascular and postural responses to nGVS.

Importantly, while other forms of vestibular stimulation, such as sinusoidal GVS, have been reported to modulate blood pressure and heart rate variability [8,14], our findings, together with previous work [10], suggest that nGVS does not provoke hemodynamic changes. The absence of cardiovascular effects represents a key advantage for clinical application, particularly in older adults or patients with neurological disorders who are often vulnerable to autonomic instability. Thus, nGVS may offer a unique combination of postural benefits with cardiovascular safety, supporting its potential use in rehabilitation settings for populations with impaired balance.

Several limitations should be acknowledged. First, this was a pilot study with a relatively small sample of healthy young adults, which limits generalizability. Second, blood pressure was assessed by automated sphygmomanometry immediately after each trial, rather than by continuous monitoring, which reduces sensitivity to transient changes. Third, the baseline was measured only once, which may increase measurement variability. Finally, the effects of nGVS may differ in populations with autonomic dysfunction or balance impairment, such as elderly individuals with orthostatic hypotension or patients with spinocerebellar degeneration.

## Conclusions

In conclusion, this pilot study demonstrated that 1 mA nGVS during quiet standing is safe and well tolerated in healthy young adults, producing no detectable changes in blood pressure or postural sway. While exploratory associations between cardiovascular and postural responses were not supported in mixed-model analysis, the consistent absence of adverse hemodynamic effects highlights the cardiovascular safety of nGVS and underscores the need for further studies in clinical populations.
